# An Innovative Formula for Keratosis Pilaris Treatment—A Randomized Controlled Study Based on the “Exfoliation‐Dissolution‐Repair” Concept

**DOI:** 10.1111/jocd.70605

**Published:** 2025-12-24

**Authors:** Fangzhou Liu, Lu Cheng, Jie Yang, Tuowei Li, Jing Li, Xiaojie Zhang, Fengwei Qi, Yuanbai Li, Yang Yang, Yexiang Zhang, Hong Meng

**Affiliations:** ^1^ Institute of Information on Traditional Chinese Medicine China Academy of Chinese Medical Sciences Beijing People's Republic of China; ^2^ Shandong Huawutang Biotechnology Co. Ltd. Ji'nan People's Republic of China; ^3^ South China University of Technology Guangzhou People's Republic of China; ^4^ EviSkin Testing Technology (Beijing) Co. Ltd. Beijing People's Republic of China; ^5^ International School of Cosmetics Beijing Technology and Business University Beijing People's Republic of China

**Keywords:** follicular papules, keratosis pilaris, papain, plant oils, scrub

## Abstract

**Objective:**

Keratosis pilaris (KP) is a common follicular keratotic skin disorder characterized by follicular papules and skin roughness, often accompanied by itching and inflammation, significantly affecting appearance and psychological health.

**Methods:**

This 28‐day prospective, open‐label, randomized controlled study evaluated the effectiveness of a novel combined formula for KP treatment. The formula included a scrub ( *Olea europaea* shell powder, hydrated silica, and papain) and a moisturizing lotion with plant oils (peony seed oil, oat kernel oil, and rice bran oil). Sixty KP patients aged 18–35 were randomly assigned to a control group (salicylic acid body lotion) or a study group (combined formula), with 30 participants each. Evaluations included follicular papule counts, transepidermal water loss (TEWL), and depression scale scores at baseline (D0), day 7 (D7), day 14 (D14), day 21 (D21), and day 28 (D28). After D28, a self‐assessment questionnaire was used to compare treatment outcomes.

**Results:**

According to the repeated‐measures ANOVA, the control and study group had a significant reduction in follicular papule counts over time (all *p* < 0.05). For TEWL values, a significant simple effect of time was observed in the study group (*p* = 0.026), whereas the control group showed only a marginal change (*p* = 0.054). Despite the absence of a significant main group effect (*p* = 0.287), these results suggest that the study group achieved a greater overall improvement in skin barrier function over time. In the self‐assessment, 21 out of 23 indicators had higher effectiveness rates in the study group than the control group, with 96.7% effectiveness in improving skin dryness, smoothness, roughness, freshness, oiliness, and oil control. Depression scale scores were significantly affected by both time and group (*p* = 0.017 and *p* = 0.008, respectively), indicating psychological improvement after 28 days.

**Conclusion:**

The combined formula effectively reduced follicular papules, improved skin hydration, decreased TEWL, and alleviated depressive symptoms in KP patients.

## 1 Introduction

Keratosis pilaris (KP) is a common follicular keratinization disorder characterized by keratin plugs in the follicular infundibulum [1], leading to skin texture roughness and follicular papules on the skin. KP is often accompanied by itchiness and skin barrier function disruption [2], further exacerbating skin dryness and irritation. The prevalence of KP is higher in adolescence, and the symptoms of the disease might resolve naturally with age in some patients [3], yet about 40% of adults continue to suffer from KP [4]. In addition, the visible skin abnormalities of KP can negatively impact patients' mental health and affect their quality of life. In adults, factors such as environment, poor dietary habits (i.e., vitamin A deficiency [5]), and endocrine disorders (e.g., androgens [6]) can further exacerbate the KP symptoms, leading to problems in daily life and social interactions. Since KP may persist long‐term and affect patients' appearance and comfort, its treatment is an important part of skincare and a critical tool to improve patients' physical and mental health. Currently, the therapeutic strategies for KP primarily focus on keratin exfoliation [7], skin moisturization, and skin barrier function restoration, with two main directions: exfoliation and moisturization. Exfoliation methods usually involve keratin softeners (e.g., salicylic acid) or mechanical/laser‐assisted exfoliation techniques [8, 9]. However, the efficacy of these treatments varies among individuals. For example, in the case of chemical peeling, although using alpha hydroxy acids (AHAs) or salicylic acids is highly effective and shows promising results, the acids are highly stimulating on the skin and may be associated with significant side effects such as temporary erythema, swelling, tingling sensation, and even hyperpigmentation and scarring, which may reduce patients' compliance with the treatment. Therefore, a highly effective, safe, and sustainable topical treatment option with minimum side effects and improved KP's therapeutic efficacy [10] is in great demand.

In recent years, the combined treatment regimens using scrubs and moisturizing lotions have garnered increasing attention. This approach is consistent with the dual objectives of KP treatment—exfoliation and skin barrier repair. Scrub granules facilitate physical exfoliation, remove keratin buildup, and thus improve skin texture; moisturizing lotion provides deep hydration to relieve skin dryness and strengthen the skin barrier. Studies have shown that such combined regimens significantly reduce skin roughness and improve erythema and pigmentation around hair follicles. In this study, a 28‐day prospective, open‐label, randomized controlled study was conducted to evaluate the efficacy of the combined formula in improving follicular papules and skin hydration in KP patients aged 18–39. The combined formula follows the skincare concept of “exfoliation‐dissolution‐repair” and is composed of a scrub containing natural *Olea europaea* shell powder, hydrated silica, and papain, and a moisturizing lotion containing plant‐based oils (peony seed oil, oat kernel oil, and rice bran oil). Meanwhile, we explored the comprehensive effect of the combined formula in improving the skin appearance and mental health of KP patients, providing scientific evidence for the holistic management of KP.

## 2 Materials and Methods

### 2.1 Clinical Patient Recruitment

Ethical approval for this study was obtained from the Ethics Committee of Beijing Technology and Business University (Approval Number: 2024‐131).

All enrolled volunteers met the diagnostic, inclusion, and exclusion criteria. Participants were informed about the purpose and procedures of the study, and written informed consent was obtained prior to their participation.

### 2.2 Diagnostic Criteria

All volunteers met the diagnostic requirements for keratosis pilaris as outlined in clinical dermatology guidelines and were confirmed as having keratosis pilaris.

### 2.3 Inclusion and Exclusion Criteria

The inclusion and exclusion criteria displayed in Table 1. Volunteers were eligible for the study if they met the inclusion criteria and were excluded from the study if they met the exclusion criteria.

**TABLE 1:** Inclusion and exclusion criteria for volunteers.

Inclusion criteria	Exclusion criteria
Were Chinese participants aged 18–35 years Diagnosed with keratosis pilaris Willing to undergo treatment, complete the entire treatment course, attend follow‐up visits on schedule, and provide written informed consent Approved by the Ethics Committee of Beijing Technology and Business University (Approval Number: 2024‐131)	Had active viral diseases, such as herpes simplex or warts, at the treatment site Had undergone cryotherapy, radiation therapy, or surgery in the past 6 months Had taken oral corticosteroids within the past 3 months Had immune deficiency diseases or were prone to scarring Were currently participating in or had participated in another clinical study/treatment within the past 3 months Were pregnant, breastfeeding, or planning pregnancy

### 2.4 Treatment Method

The study observation period lasted 28 days. Participants applied the assigned samples according to the prescribed method and frequency. Follow‐up assessments were conducted on Day 7 (D7), Day 14 (D14), Day 21 (D21), and Day 28 (D28), during which primary and secondary outcomes were evaluated. At the end of the treatment, participants completed a questionnaire to assess their perceived improvements. The entire study was conducted strictly according to the clinical trial protocol to ensure data accuracy and completeness. Details of the test samples are listed in Table 2.

**TABLE 2:** Test samples information.

Group	Sample name	Batch number
Control group	CeraVe salicylic acid body lotion	20Y110
Study group	Banmuhuatian ice cream smooth scrub (scented)	20240823
	Banmuhuatian essential oil moisturizing body lotion (rose garden scent)	FG26072ABRG

*Note:* The study group used a two‐step combined regimen consisting of a body scrub (exfoliation step) followed by a moisturizing body lotion (repair step). The detailed application method is described in Sections 2.4.1 and 2.4.2.

*Note:* Before beginning of the trial, a one‐week washout period was established, that is, participants adhered to regular showering habits and used standard cleansing products without any body care products (e.g., scrubs or body lotions). Throughout the study, participants were required to maintain consistent showering routines and use the same cleansing products.

#### 2.4.1 Control Group

Participants applied salicylic acid body lotion to the designated area once daily, massaging until fully absorbed.

#### 2.4.2 Study Group

Participants applied the scrub to both upper arms, covering the entire area, and massaged gently for 20–30 s. After rinsing thoroughly and drying, the moisturizing lotion was applied to both arms, using at least one pump per arm, and massaged until fully absorbed.

### 2.5 Observation Indicators and Test Instruments

Observations of skin conditions were conducted on D0 (baseline), D7, D14, D21, and D28. The primary observation indicators included: follicular papule count and skin hydration parameters. All tests were performed by professionally trained technicians to ensure data consistency and accuracy. The testing environment was controlled at a temperature of 20°C–22°C and humidity of 40%–60%. All devices were calibrated prior to each session to ensure data reliability.

#### 2.5.1 DHS Skin Health System

The follicular papule was detected and quantified using DHS Skin Health System (DHS2100, Beijing Jingchenggongfang Electronic Integration Technology Co. Ltd., China).

The DHS Skin Health System is a high‐magnification dermatoscope, similar in function to otoscopes or ophthalmoscopes used in ENT practice. This device is an effective tool for observing skin pigmentation disorders. In this study, the system was used to capture skin images, record follicular papule counts, and calculate the follicular papule improvement index.

#### 2.5.2 Aqua Flux AF200

The skin hydration content was measured by Aqua Flux AF200 (BIOX, UK).

The Aqua Flux can measure the changes in water vapor pressure on the skin surface to infer the transepidermal water loss (TEWL) in g/h/m^2^ (grams of water lost per hour per square meter of skin).

### 2.6 Skin Condition Improvement Questionnaire and Effectiveness Rate

After the 28‐day treatment period, a questionnaire assessing the effectiveness of the skin condition improvements was conducted (Table 3). The calculation of effectiveness is based on the effective individuals (the sum of participants selecting “agree” and “strongly agree” on the relevant indicator) and total number of respondents. Self‐assessed effectiveness rate formula is: Self‐assessed effectiveness rate (%) = (number of effective individuals/total number of respondents) × 100%.

**TABLE 3:** Self‐assessed questionnaire contents and description.

Indicator	Description
Relief of dry skin	Assessment of perceived relief in skin dryness
Skin feels more hydrated and delicate	Evaluation of skin hydration and texture improvement
Skin feels smoother	Perception of smoother skin after treatment
Improvement in skin roughness	Reduction in overall skin roughness
Better skin tolerance	Improved ability of skin to tolerate the product
Enhanced skin barrier function	Strengthening of the skin's protective barrier
Reduction of scaling on upper arms	Decrease in visible scaling on treated areas
Improvement in redness on upper arms	Reduction in redness on the treated areas
Reduction in itchiness on upper arms	Relief from itchiness on treated areas
Relief of pain on upper arms	Pain reduction in the affected areas
Reduction of dead skin on upper arms	Decrease in accumulated dead skin cells
Product effectively removes body oil	Effectiveness of product in reducing oiliness
Skin feels fresher overall	Perception of a fresher and cleaner skin feel
Longer‐lasting feeling of freshness	Duration of the feeling of skin freshness
Reduced body oil production	Improvement in reducing body oil secretion
Product has an oil‐control effect	Effectiveness in controlling excess oil production
Improvement in overall oiliness	Perception of improvement in overall oiliness
Reduction in follicular papules on arms	Decrease in visible follicular papules
Slower formation of new follicular papules	Reduced rate of new follicular papule formation
Improvement in severity of follicular papules	Reduction in severity of existing follicular papules
Product suitability for follicular papules	Suitability of the product for managing follicular papules
Relief of discomfort associated with follicular papules	Relief from discomfort linked to follicular papules
Product is gentle and non‐irritating	Perception of product gentleness and lack of irritation

### 2.7 Depression Scale Test

The BECK Depression Inventory (BDI) was used to assess the psychological impact of the combined formula on KP patients at D0, D7, D14, D21, and D28. The evaluation criteria for depression scores were as follows:

- 0–5: Normal state.
- 6–15: Mild depressive symptoms.
- 16–25: Moderate depressive symptoms.
- 26–45: Severe depressive symptoms.

### 2.8 Statistical Analysis

Descriptive statistical analysis was performed for each indicator, and data were analyzed using SPSS version 26.0 (IBM Corp., Armonk, NY, USA).

Descriptive statistics (mean ± standard deviation) were calculated for all continuous variables. Data were tested for normality using the Shapiro–Wilk test. To account for repeated observations over time and to control for type I error, a repeated‐measures ANOVA was applied to examine the main effects of time and treatment group, as well as their interaction. When significant effects were observed, LSD post hoc tests and simple main effect analyses were conducted to identify specific differences between time points or groups. All statistical analyses were conducted using two‐tailed tests, with a significance level of *α* = 0.05\alpha = 0.05. A *p*‐value < 0.05 was considered statistically significant.

## 3 Demographics and Results

According to the diagnostic, inclusion, and exclusion criteria, a total of 60 volunteers with keratosis pilaris (KP) were enrolled in this study (Table 4).

**TABLE 4:** Participant demographics.

Variable	Control group (*n* = 30)	Study group (*n* = 30)
Gender, *n* (%)		
Female	23 (76.7%)	20 (66.7%)
Male	7 (23.3%)	10 (33.3%)
Age (years, mean ± SD)	25.4 ± 6.3	25.4 ± 5.4
Median age (years, minimum and maximum range)	22.5 (18.0, 35.0)	25.0 (19.0, 35.0)

The study aimed to evaluate the effectiveness of the combined formula in improving KP. It was approved by the Ethics Committee of Beijing Technology and Business University (Approval Number: 2024‐131), and conducted in accordance with Good Clinical Practice (GCP) guidelines and the Declaration of Helsinki.

**FIGURE 1:**
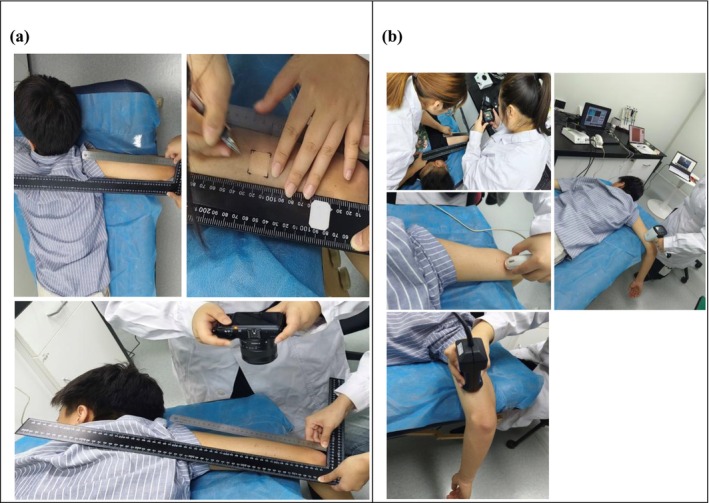
Representative macroscopic photographs of the lateral upper arm test area taken during the study. (a) Site marking by a dermatologist to identify the KP region prior to measurement. (b) Standardized imaging and measurement procedure using the DHS Skin Health System and Aqua Flux AF200.

Before measurement, the dermatologist confirmed the presence and location of KP lesions on the lateral upper arm. The target area was marked using a non‐permanent medical skin marker to ensure that subsequent evaluations were performed on the same region. Representative macroscopic photographs were taken before each test session under standardized lighting and fixed camera distance to maintain consistency in positioning. These visual records were archived as part of the study documentation to support data verification, methodological transparency, and ethical review compliance. Figure 1 provides an macroscopic overview of the test area.

**FIGURE 2:**
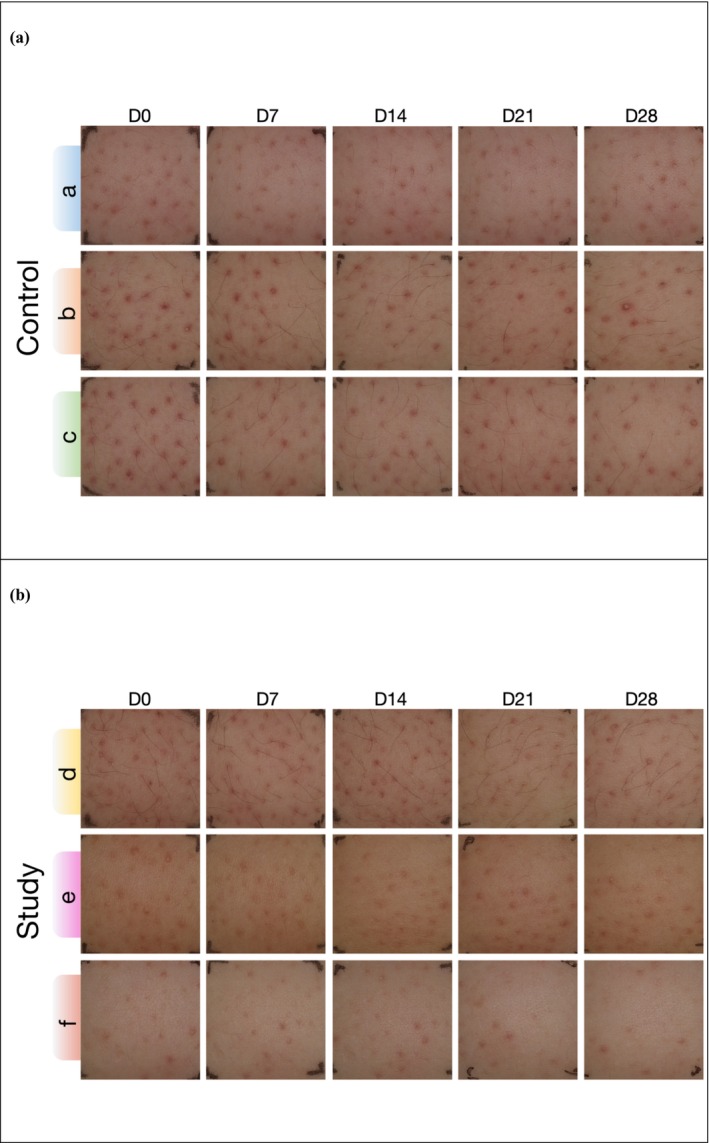
Representative visual in skin condition from three participants in each group at five time points (D0, D7, D14, D21, and D28). (a) Control group. (b) Study group.

The results showed that the combined formula effectively reduced the number of follicular papules and decreased transepidermal water loss (TEWL), while also alleviating depression symptoms and supporting the psychological health of participants. Figure 2 illustrates the progressive improvements in skin condition among three participants in the control (a) and study (b) group at five time points (D0, D7, D14, D21, and D28). The test sites were located on the upper arms of both sides for all participants.

### 3.1 Follicular Papule Assessment

Follicular papule assessment was based on the count of follicular papules. According to the Shapiro–Wilk test, all data were normally distributed across groups. Mauchly's test of sphericity indicated that the assumption of sphericity was violated (*W* = 0.162, *p* < 0.001) and the results were corrected using the Greenhouse–Geisser method.

A repeated‐measures ANOVA showed that the main effect of group was not significant (*p* = 0.106), indicating that there was no overall difference in follicular papule counts between the control and study groups. The main effect of time was significant (*p* < 0.001), suggesting that follicular papule counts changed significantly across different time points within the same group. The interaction between time and group was significant (*p* = 0.021), indicating that the change pattern over time differed between the control and study groups.

Univariate tests showed no between‐group differences were found at any single time point (*p* = 0.054, 0.124, 0.101, 0.209, and 0.139). Multivariate tests of simple effects showed that time had a significant effect in both the control group (*p* < 0.001) and the study group (*p* < 0.001), indicating significant within‐group improvements over time. Pairwise comparisons demonstrated that, in the control group, follicular papule counts decreased significantly from baseline (D0) to each subsequent measurement time point (all *p* < 0.001, except for D14 to D21 that had *p* = 0.010). In the study group, significant differences were found among all time points from baseline (D0) to each subsequent measurement time point (all *p* < 0.001, except for D21 to D28 that had *p* = 0.007) (Table 5).

**TABLE 5:** Follicular papule count (mean ± SD) over time in the control and study groups analyzed by repeated‐measures ANOVA.

Indicator	Group	Baseline (D0)	D7	D14	D21	D28	Repeated Measures ANOVA		
							*F*	*p*	*η* ^2^
Follicular papule count	Control	5.37 ± 2.53^a^	4.83 ± 2.31^b^	4.37 ± 2.06^c^	4.03 ± 2.02^d^	3.64 ± 1.80^e^			
	Study	6.80 ± 3.07^a^	5.86 ± 2.77^b^	5.39 ± 2.63^c^	4.70 ± 2.05^d^	4.40 ± 2.07^e^			
Group main effect							2.697	0.106	0.044
Time main effect							121.39	< 0.001	0.677
Group*Time interaction							4.036	0.021	0.065

*Note:* Different superscript letters “a, b, c, etc.” within the same row indicate significant differences between time points (*p* < 0.05, LSD post hoc test).

**FIGURE 3:**
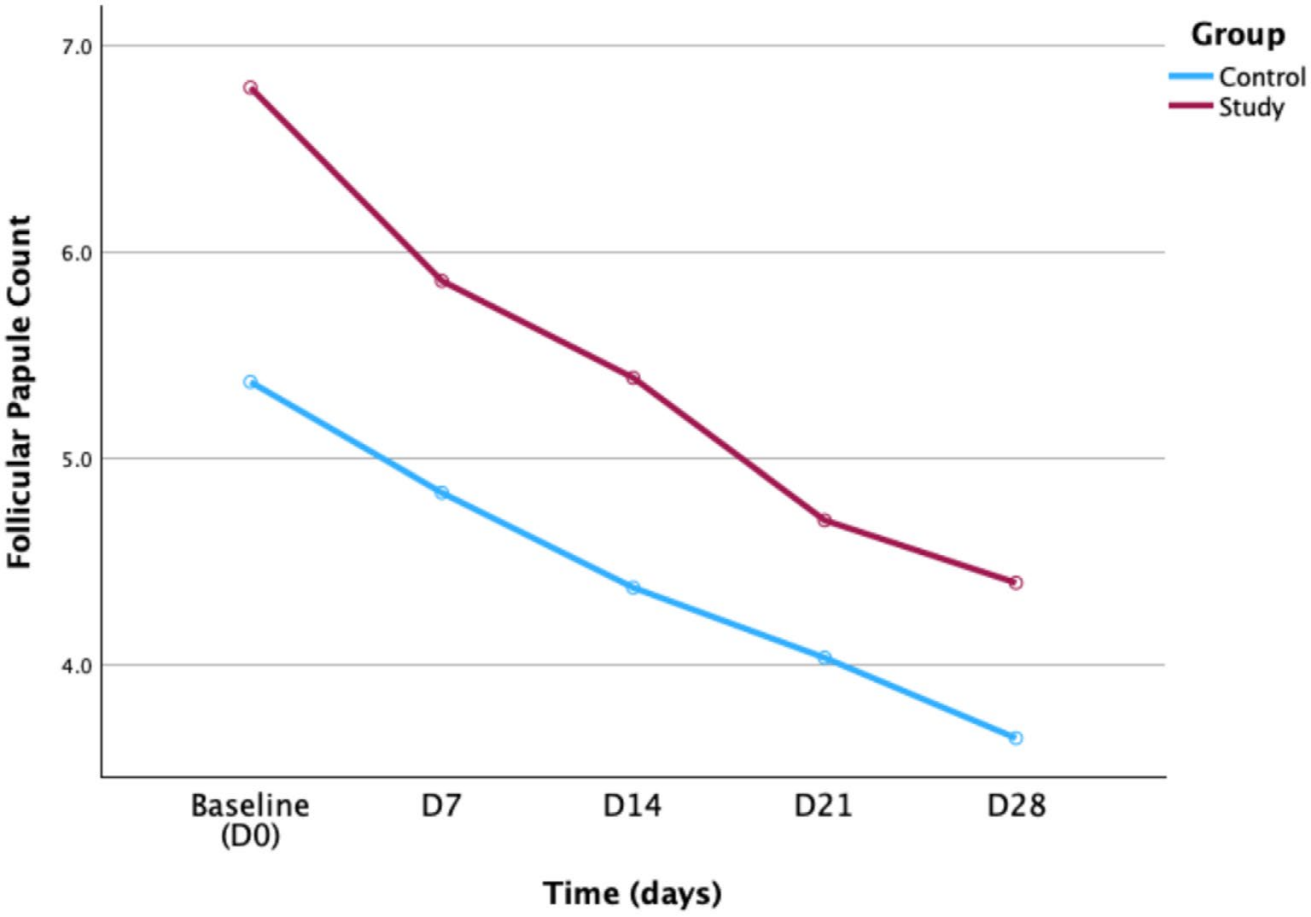
Changes in follicular papule count over time in the control and study groups.

Figure 3 showed the trend in follicular papule counts over time between the control and study groups.

### 3.2 Skin Hydration Assessment

The skin hydration assessment was shown by transepidermal water loss (TEWL) value. According to the Shapiro–Wilk test, all data were normally distributed across groups. Mauchly's test of sphericity indicated that the assumption of sphericity was met (*W* = 0.816, *p* = 0.243). No further correction was required.

A repeated measures ANOVA showed no significant main effect of group (*p* = 0.287), indicating that the TEWL values did not differ significantly between the control and study groups. The main effect of time was significant (*p* = 0.003), indicating that the TEWL values changed significantly across different time points within the same group. The interaction between time and group was not significant (*p* = 0.817), implying that both groups exhibited a similar trend of change over time (Table 6).

**TABLE 6:** TEWL values (mean ± SD) over time in the control and study groups analyzed by repeated‐measures ANOVA.

Indicator	Group	Baseline (D0)	D7	D14	D21	D28	Repeated measures ANOVA		
							*F*	*p*	*η* ^2^
TEWL (g/m^2^h)	Control (*n* = 30)	9.33 ± 4.33^a^	9.54 ± 3.74^a^	9.66 ± 4.09^a^	9.77 ± 2.95^b^	8.48 ± 2.93^c^			
	Study (*n* = 30)	8.35 ± 2.92^a^	8.68 ± 2.89^a^	8.55 ± 2.68^a^	9.50 ± 3.41^a^	7.84 ± 2.85^b^			
Group main effect							1.153	0.287	0.019
Time main effect							4.072	0.003	0.066
Group*Time interaction							0.388	0.817	0.007

*Note:* Different superscript letters “a, b, c, etc.” within the same row indicate significant differences between time points (*p* < 0.05, LSD post hoc test).

Univariate test results revealed that no between‐group differences were observed at any single time point (*p* = 0.309, 0.327, 0.220, 0.748, and 0.395). Regarding multivariate test, the simple effect of time was not significant in the control group (*p* = 0.054) but was significant in the study group (*p* = 0.026), indicating a time‐dependent improvement in TEWL within the study group. Pairwise comparisons showed that, in the control group, significant differences were observed between D7 versus D28 (*p* = 0.033), D14 versus D28 (*p* = 0.015), and D21 versus D28 (*p* = 0.008), suggesting that the improvement mainly occurred during the final stage of the trial. In the study group, a significant difference was found between D21 and D28 (*p* < 0.001).

**FIGURE 4:**
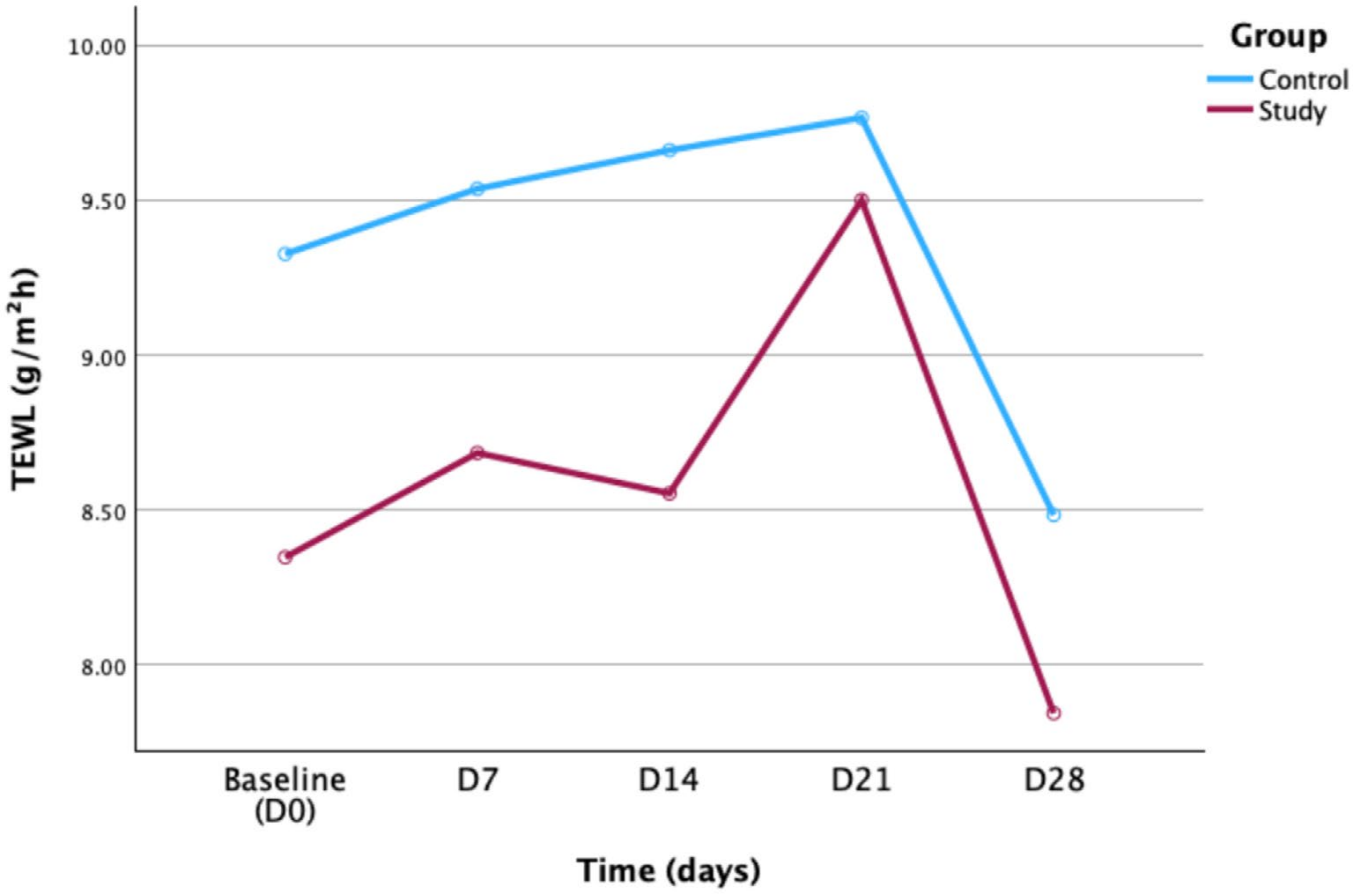
Changes in TEWL values over time in the control and study groups.

Figure 4 showed the trend of TEWL values over time between the control and study groups.

### 3.3 Skin Condition Improvement Effectiveness Rate

After 28 days of treatment with the combined formula, 21 out of 23 indicators in the study group had higher self‐assessed effectiveness rates than the control group, except for indicators “better skin tolerance” and “product is gentle and non‐irritating” (Table 7).

**TABLE 7:** Self‐assessed skin condition improvement effectiveness rate.

Indicator	Control group (%)	Study group (%)
Relief of dry skin	93.30	96.70
Skin feels more hydrated and delicate	90.00	96.70
Skin feels smoother	90.00	96.70
Improvement in skin roughness	86.70	96.70
Better skin tolerance	83.30	76.70
Enhanced skin barrier function	83.30	86.70
Reduction of scaling on upper arms	83.30	86.70
Improvement in redness on upper arms	80.00	93.30
Reduction in itchiness on upper arms	76.70	80.00
Relief of pain on upper arms	70.00	73.30
Reduction of dead skin on upper arms	83.30	90.00
Product effectively removes body oil	60.00	90.00
Skin feels fresher overall	63.30	96.70
Longer‐lasting feeling of freshness	66.60	83.30
Reduced body oil production	56.70	96.70
Product has an oil‐control effect	53.40	96.70
Improvement in overall oiliness	60.00	83.30
Reduction in follicular papules on arms	80.00	90.00
Slower formation of new follicular papules	76.70	76.70
Improvement in severity of follicular papules	73.30	83.30
Product suitability for follicular papules	83.40	93.30
Relief of discomfort associated with follicular papules	86.70	93.30
Product is gentle and non‐irritating	93.30	90.00

### 3.4 Depression Scale Score Assessment

To evaluate the psychological impact of the combined formula on KP patients, the study utilized the Beck Depression Inventory (BDI). According to the Shapiro–Wilk test, all data were normally distributed across groups. Mauchly's test of sphericity indicated that the assumption of sphericity was violated (*W* = 0.264, *p* < 0.001) and the results were corrected using the Greenhouse–Geisser method.

A repeated measures ANOVA showed significant main effects of group (*p* = 0.008), indicating that the depression scale score differed significantly between the control and study groups. The main effect of time was significant (*p* = 0.017), indicating that the depression scale score changed significantly across different time points within the same group. The interaction between time and group was not significant (*p* = 0.103), implying that both groups exhibited a similar trend of change over time (Table 8).

**TABLE 8:** Depression scale scores (mean ± SD).

Indicator	Group	Baseline (D0)	D7	D14	D21	D28	Repeated measures ANOVA		
							*F*	*p*	*η* ^2^
Depression scale score	Control	4.37 ± 5.26^a^	4.97 ± 7.22^a^	5.07 ± 5.28^a^	3.80 ± 4.87^a^	4.17 ± 5.91^a^			
	Study	5.40 ± 4.89^a^	4.07 ± 5.86^ab^	2.83 ± 3.72^b^	0.80 ± 1.38^c^	0.67 ± 1.58^c^			
Group main effect							7.645	0.008	0.116
Time main effect							3.699	0.017	0.060
Group*Time interaction							2.162	0.103	0.036

*Note:* Different superscript letters “a, b, c, etc.” within the same row indicate significant differences between time points (*p* < 0.05, LSD post hoc test).

Univariate tests of simple effects showed that between‐group differences were not significant at baseline (D0), D7, or D14 (*p* = 0.434, 0.598, and 0.063, respectively), but became significant at D21 and D28 (*p* = 0.002 and 0.003). For the simple effects of time, no significant differences were found across time points within the control group (*p* = 0.604), whereas the study group showed a significant time effect (*p* = 0.002). Pairwise comparisons detected no significant pairwise differences in the control group. Within the study group, the depression scale score decreased significantly between baseline (D0) and D14 (*p* = 0.029), D21 (*p* < 0.001), and D28 (*p* < 0.001), between D7 and D21 (*p* = 0.028) and D28 (*p* = 0.037), and between D14 and D21 (*p* = 0.017) and D28 (*p* = 0.024).

**FIGURE 5:**
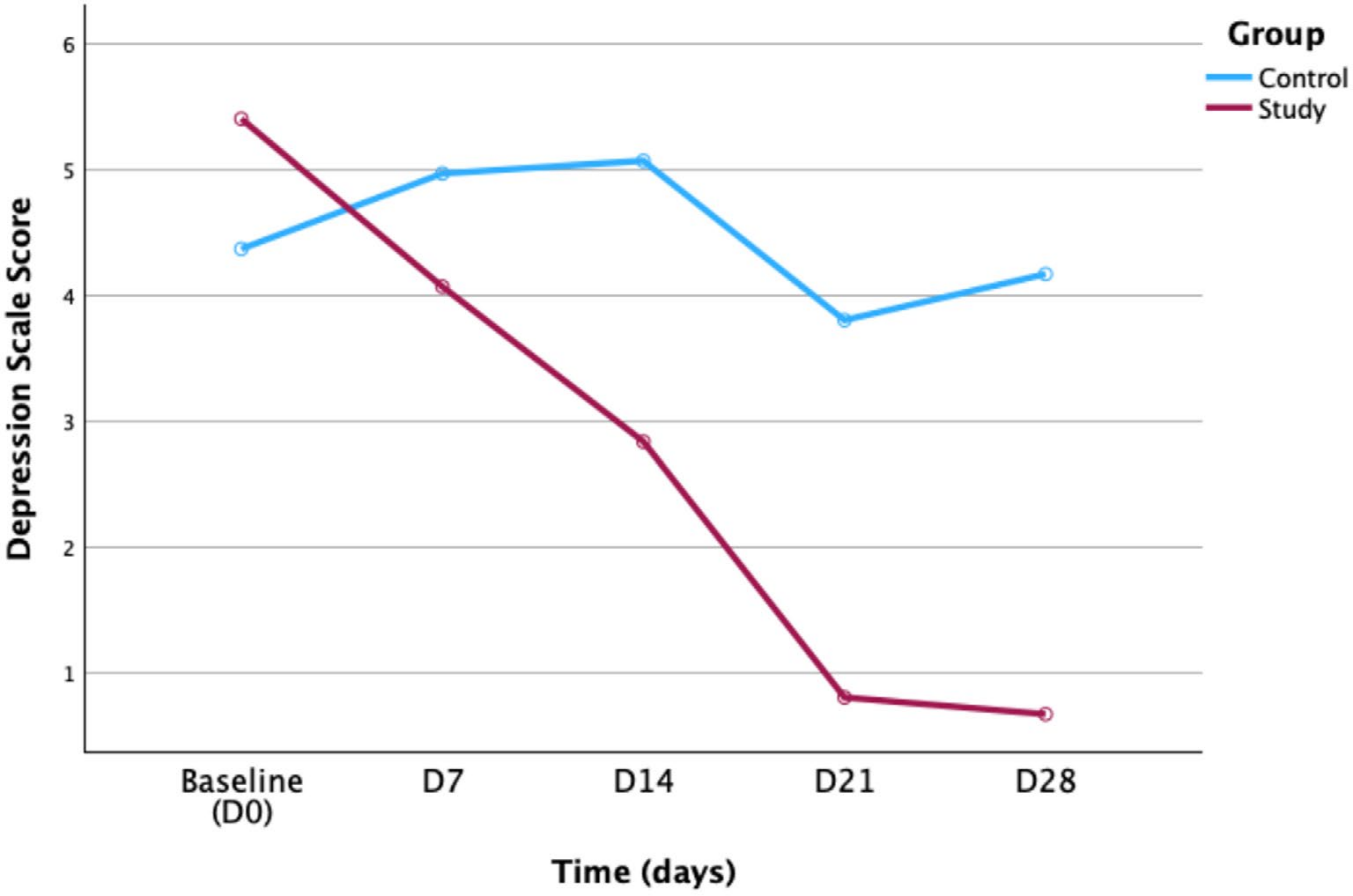
Changes in depression scale scores over time in the control and study groups.

Figure 5 showed the trend of depression scores over time between the control and study groups.

## 4 Discussion

Keratosis pilaris (KP) is mainly caused by keratin embolism, characterized by distinctive keratinized follicular papules and skin roughness [11]. These symptoms may negatively affect patients' appearance and mental health. As a chronic keratosis pilaris skin disease, KP may persist throughout a patient's lifetime, making it an important health concern for adult populations. Due to the challenges of treating KP, the current topical KP treatment options are relatively homogenous [12], without effective, comprehensive therapeutic strategies targeting keratolytic exfoliation, dissolution, skin moisturization, and anti‐inflammation. Therefore, it is crucial to develop a combined and efficient treatment process.

The study demonstrated that the combined formula significantly improved KP patients' skin symptoms. After 28 days of use, the number of follicular papules in the control and study group was significantly reduced compared to the baseline. From the skin hydration assessment, although no significant between‐group differences were found at any specific time point, multivariate analysis demonstrated a significant time effect in the study group (*p* = 0.026) but not in the control group (*p* = 0.054). This indicates that the study group experienced a more consistent decline in TEWL over the 28‐day treatment period and there was a significant improvement in skin barrier function. The self‐assessed questionnaires showed that apart from the “better skin tolerance” and “product is gentle and non‐irritating,” there were 21 indicators with higher self‐assessed efficiency rates in the study group than in the control group. Notably, in “relief of dry skin,” “skin feels smoother,” “improvement in skin roughness,” “skin feels fresher overall,” “reduced body oil production,” and “product has an oil‐control effect” indicators, the self‐assessed efficiency rate reported 96.7% in the study group. The results of the depression scale assessment revealed a significant reduction in BDI scores in the study group, particularly from D14 to D28, while the control group showed no statistically significant change across time. The significant time effect within the study group (*p* = 0.002) and the between‐group differences observed at D21 and D28 (*p* = 0.002 and 0.003) indicated that the combined exfoliation‐moisturizing formula effectively alleviated depressive symptoms associated with KP. These findings confirmed the multi‐functional efficacy of the combined formula in KP care.

**FIGURE 6:**
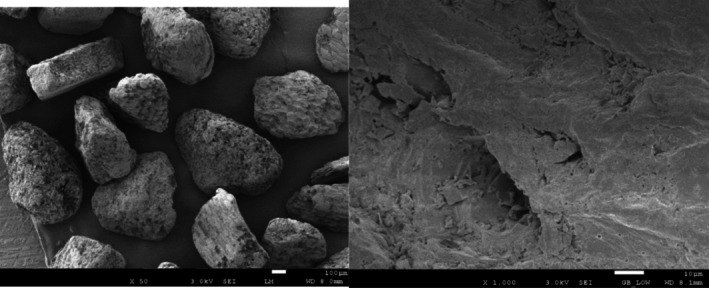
SEM image of exfoliating particles ( *Olea europaea* shell powder).

**FIGURE 7:**
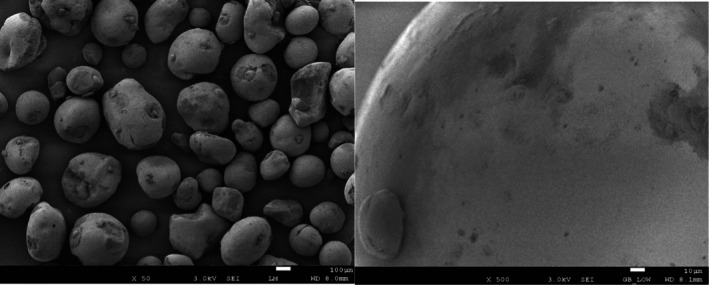
SEM image of exfoliating particles (hydrated silica).

Based on the concept of “exfoliation‐dissolution‐repair” [13], this study conducted a combination of physical scrub ( *O. europaea* shell powder and hydrated silica) and chemical exfoliation (papain). The natural *O. europaea* shell powder and hydrated silica formed physical exfoliation particles derived from plant and chemical sources [14, 15]. From the scanning electron microscopy (SEM) image, *O. europaea* shell particles appeared as irregular block structures with a dense surface and a particle size ranging from 100 to 500 μm (Figure 6). Hydrated silica particles were spherical with dense surfaces, and their size ranged from 100 to 600 μm, with uneven distribution (Figure 7). By optimizing the blend of exfoliation particles, the scrub balanced softness and hardness, effectively and safely removing keratin buildup without over‐irritating the skin. Papain was selected as a mild alternative to acidic agents for chemical exfoliation. It utilized gentle keratolysis by its protein hydrolysis function to break down the degraded and waste stratum corneum and accelerate cellular metabolism [16, 17]. The dual exfoliation mechanism efficiently removes keratin buildup while maintaining skin gentleness, enhancing the absorption of plant oils (peony seed oil, oat kernel oil, and rice bran oil) in the moisturizing lotion, providing deep moisturization in the skin, and assisting in the skin barrier repairment [18].

The moisturizing lotion contained three primary plant oils: peony seed oil, oat kernel oil, and rice bran oil. Peony seed oil is rich in polyphenols (e.g., paeoniflorin, baicalein) and a variety of fatty acids (e.g., linoleic acid, linolenic acid), which provides strong antioxidant activity, forms a protective lipid barrier (that can reduces water loss and moisturizes and repairs the skin), and promotes cell regeneration; the topical application of peony seed oil protects the skin and reduces UV‐induced damage [19]. Oat kernel oil contains linoleic acid (36%–46%), oleic acid (28%–40%), and oat alkaloids as antioxidant and anti‐inflammatory components. It increases ceramide levels in keratinocytes, contributes to skin barrier repair, and alleviates skin dryness and itching [20, 21, 22]. Rice bran oil includes γ‐glutamine (γ‐oryzanol), tocopherols, and tocotrienols, which offer antioxidant, anti‐inflammatory and barrier‐repairing effects [23, 24].

In summary, the combined formula significantly improved KP symptoms among patients aged 18–39. It effectively reduced follicular papules, decreased transepidermal water loss, and enhanced patients' psychological satisfaction by alleviating depressive symptoms. These findings suggest that the combined formula is clinically valuable for managing KP. However, the study had certain limitations. First, the sample size was relatively small (30 participants per group and 60 in total), which may limit the statistical efficacy. Second, the 28‐day observation period primarily assessed short‐term efficacy. The combined formula's long‐term effects were not evaluated comprehensively, and no post‐discontinuation follow‐up was conducted. Participants may have resumed their previous skincare products after the trial, which could influence long‐term outcomes. Future studies should include larger sample sizes and extended follow‐up periods (e.g., at 4, 8, and 12 weeks after discontinuation) to fully assess the combined formula's sustained long‐term patient satisfaction and adherence and further validate this study's results.

## Author Contributions

F.L. and L.C. are co‐first authors and contribute equally to this work. L.C., J.Y., F.Q., Y.Z., and H.M. performed the research. F.L., Y.L., and Y.Y. contributed to the conceptualization and data curation. J.L., X.Z., and Y.L. worked on investigation and validation of the data. F.L. and Y.Y. worked on supervision and visualization. F.L. and T.L. wrote the original draft; L.C., T.L., Y.Y., and Y.L. reviewed and edited the paper.

## Funding

This work was supported by the National Natural Science Foundation Youth Program (Grant 82204780), the Major Breakthrough Project of the Science and Technology Innovation Project of the Chinese Academy of Chinese Medical Sciences (Grant CI2021A05311), the Special Program for Training Excellent Young Science and Technology Talents of the Chinese Academy of Traditional Chinese Medicine (Grant ZZ15‐YQ‐052), and the Key Laboratory of Multi‐source Digital and Intelligent Traditional Chinese Medicine Innovation, IICTM, CACMS (Grant ZZSYS‐1902‐01CZ).

## Ethics Statement

This study was approved by the Ethics Committee of Beijing Technology and Business University (Approval Number: 2024‐131).

## Conflicts of Interest

The authors declare no conflicts of interest.

## Data Availability

The data supporting the findings of this study are available from the corresponding author upon reasonable request. Due to ethical and privacy considerations, the data are not publicly available.
